# Correlation between retinal vein pulse amplitude, estimated intracranial pressure, and postural change

**DOI:** 10.1038/s41526-023-00269-0

**Published:** 2023-03-31

**Authors:** W. H. Morgan, J. Khoo, A. Vukmirovic, A. Abdul-Rahman, D. An, A. Mehnert, D. Obreschkow, E. Chowdhury, D. Y. Yu

**Affiliations:** 1 Lions Eye Institute, Centre for Ophthalmology and Visual Science, University of Western Australia, Perth, WA Australia; 2 International Space Centre, 35 Stirling Hwy, Crawley, WA 6009 Australia; 3 Department of Ophthalmology, Counties Manukau DHB, Auckland, New Zealand; 4 International Centre for Radio Astronomy Research (ICRAR), M468, University of Western Australia, 35 Stirling Hwy, Crawley, WA 6009 Australia; 5 Information Technology, Murdoch University, 90 South Street, Murdoch, WA 6150 Australia

**Keywords:** Physiology, Scientific data

## Abstract

Spaceflight associated neuro-ocular syndrome (SANS) is common amongst astronauts on long duration space missions and is associated with signs consistent with elevated cerebrospinal fluid (CSF) pressure. Additionally, CSF pressure has been found to be elevated in a significant proportion of astronauts in whom lumbar puncture was performed after successful mission completion. We have developed a retinal photoplethysmographic technique to measure retinal vein pulsation amplitudes. This technique has enabled the development of a non-invasive CSF pressure measurement apparatus. We tested the system on healthy volunteers in the sitting and supine posture to mimic the range of tilt table extremes and estimated the induced CSF pressure change using measurements from the CSF hydrostatic indifferent point. We found a significant relationship between pulsation amplitude change and estimated CSF pressure change (*p* < 0.0001) across a range from 2.7 to 7.1 mmHg. The increase in pulse amplitude was highest in the sitting posture with greater estimated CSF pressure increase (*p* < 0.0001), in keeping with physiologically predicted CSF pressure response. This technique may be useful for non-invasive measurement of CSF pressure fluctuations during long-term space voyages.

## Introduction

Features of spaceflight associated neuro-ocular syndrome (SANS) develop in 38 to 51% of astronauts on long duration (more than 6 months) space flight according to the NASA HRP Evidence Report (https://humanresearchroadmap.nasa.gov/evidence/reports/SANS.pdf)^1^.

Optic nerve sheath distension is found in the majority and cerebrospinal fluid (CSF) pressure elevation in half of astronauts following return to earth^2^. Microgravity is known to prevent the usual reduction in CSF pressure when going from supine to sitting posture^3^, and has been postulated to cause a mean rise in CSF pressure over time^4^.

Spaceflight associated neuro-ocular syndrome is one of the highest priority health risks for human flights to Mars^1^. Some features of SANS such as papilloedema and choroidal folds may be due to elevated intracranial pressure (ICP) and may be detected using retinal photography. Other features such as dilated optic nerve subarachnoid space and globe flattening may not be detected in this manner and often require radiological imaging. Elevated or poorly regulated ICP is thought to be implicated in the causation of SANS, however ICP can only be measured using invasive techniques^5^.

Invasive continuous ICP monitoring requires intracranial catheter insertion via skull burr hole and intermittent ICP measurement can be performed via lumbar puncture^6–8^. However, these interventions are associated with risks and are difficult to perform in space. NASA is investigating invasive and noninvasive ICP monitoring of astronauts on the International Space Station. This motivates the development of a non-invasive technology to quantify CSF pressure change so that pressure variations in outer space can be observed during the mission. This would allow a correlation between CSF pressure fluctuations with known clinical signs of raised intracranial pressure. Additionally, it would allow the effect of interventions such as forms of artificial gravity and intermittent orbital counter-pressure to be monitored.

Techniques developed on Earth to estimate physiologic parameters in space generally require validation using a tilt table to vary posture. Cerebrospinal fluid pressure is known to vary significantly with posture, rising an average 10 mmHg from the sitting to supine posture^9^. Any potentially useful technique would need to be able to detect a change in CSF pressure over this range. Without invasive techniques, the CSF pressure change induced by postural change cannot be known with certainty. However, it can be estimated by identifying the average position of the hydrostatic indifferent point, at which location the CSF pressure remains approximately invariant under postural change. Measuring the change in vertical height from this point to the eye can give a reasonably accurate (±3 mmHg) estimate of the expected change in CSF pressure^10^. The most accurate estimate is obtained using a model that identifies the top of external jugular vein collapse, which in turn indicates the venous hydrostatic indifferent point^10^.

Retinal vein pulsation reduces as CSF pressure rises^11^. This can be detected objectively through measurement of the retinal vein pulsation amplitude using a modified form of photoplethysmography (PPG), which we have developed^12^. Retinal vein pulsation amplitudes are maximal, with greatest signal-to-noise ratios, close to the centre of the optic disc and rapidly attenuate distally along the vessel towards the optic disc rim^13^. Additionally, retinal vein pulsation amplitudes increases with raised intraocular pressure (IOP)^13^. In previous work we have used the maximal amplitudes only, with a broken stick analysis to predict intracranial pressure in a group of subjects^14^. This is valid only when a subject’s CSF pressure is close to or greater than the intraocular pressure (IOP).

In this study, we used our modified PPG technology to measure normal participants in two different postures. Due to a paucity of data in the low IOP range, the broken stick methodology was inappropriate as subjects were likely to have normal intracranial pressure^14^. Therefore, we compared pulsation amplitudes from all data points within the central half (0.4 mm radius) of the optic disc centre.

## Results

### Postural changes

**Fig. 1:**
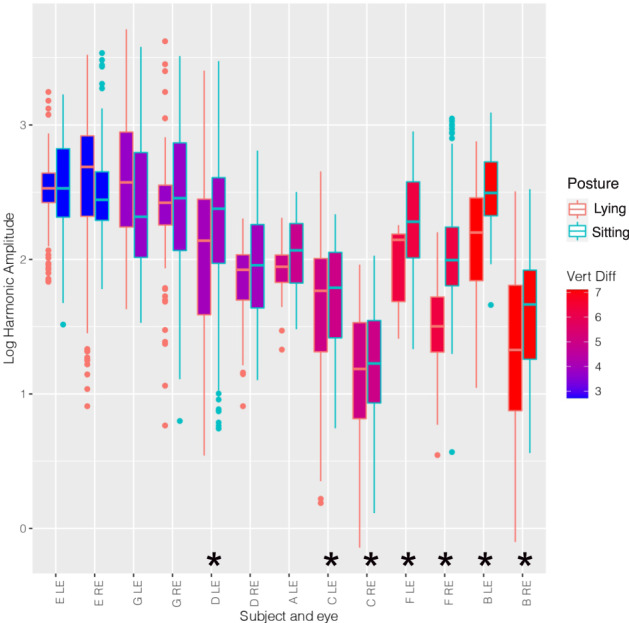
Box plot of mean log amplitudes for each eye. The data from each eye is presented in pairs representing each posture, sitting outlined in blue and lying outlined in red. The fill colour of each box represents the estimated CSF pressure difference (Vert Diff in mmHg) calculated by the postural change and is graded from blue (lowest) to red (greatest). Subjects are denoted by first letter and eye by right (RE) or left (LE). Eyes with a significant postural difference in log amplitudes are indicated with an Asterix. Centre line of the boxplot refers to the median, the lower bound of the box is the first quartile, the upper bound of the box is the third quartile, upper whisker corresponds to the third quartile plus 1.5 times the interquartile range and the lower whisker corresponds to the first quartile minus 1.5 times the interquartile range.

From 14 eyes of 7 participants, video sequences were recorded. In one eye of one subject there was inadequate video quality for analysis. So, 4801 vein pulsation amplitude data points from 13 eyes were analysed. Subjects had mean age of 42 (sd 14) years, four of whom were male. The mean baseline IOP was 14.0 mmHg (sd 4.8). The distribution of pulse amplitudes between the two postures tended to separate with increase in vertical posture difference (presumed CSF pressure difference, Fig. 1). As expected, the amplitudes in the sitting posture tended to be higher. The range of estimated CSF pressure differences was from 2.7 to 7.1 mmHg.

All hypothesis tests were performed at the 5% level of significance. The initial linear mixed model from 13 eyes of seven subjects demonstrated a significant relationship between: increasing IOP (coefficient 0.0087), increased distance from disc centre (coefficient 1.0400) and sitting (coefficient 0.1829) with increased logarithm of pulse amplitude (*p* < 0.0001). The additional linear mixed model demonstrated a significant relationship between the interaction of posture change with estimated CSF pressure difference, and the pulse amplitude (*p* < 0.0001). The logarithm of amplitude increased by 0.069 units per mmHg increase in estimated CSF pressure difference between the supine compared to sitting position. When sitting, subjects tended to have a lower pulse blood pressure than when supine, which did not achieve statistical significance (*p* = 0.1310).

### Estimated CSF pressure change associations

**Fig. 2:**
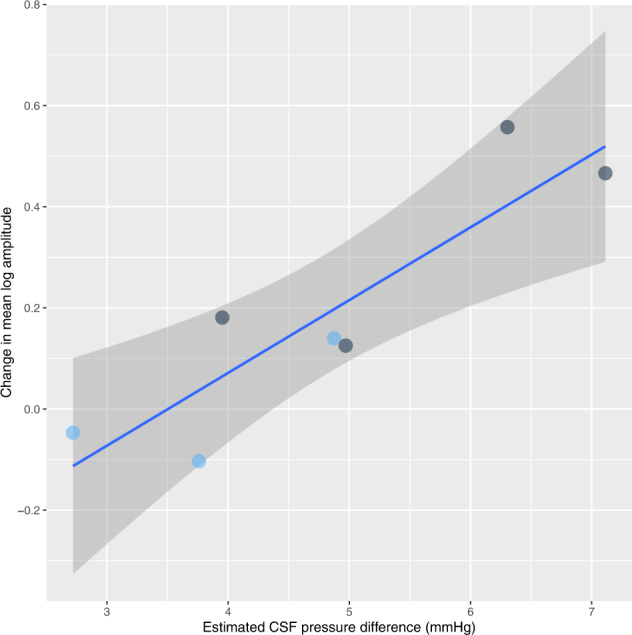
Changes between mean log amplitudes vs estimated CSF pressures. Relationship of change between mean log amplitudes of both eyes for each individual in the 2 postures (sitting–lying) and the estimated CSF pressure differences (mmHg). Points (dark grey) with significant amplitude differences between postures are shown compared to those (light blue) without significant difference. Line of best fit (solid line) is shown with 95% confidence interval.

We tested the relationship between estimated CSF pressure difference and the mean difference in logarithm of amplitudes (coefficient 0.0981, *p* = 0.0179) for each eye using a linear mixed model incorporating subject as the random factor. When data from left and right eyes were grouped to form data from each subject a similar significant relationship was identified using linear regression (slope 0.1439, *p* = 0.0072, *r* = 0.8902, Fig. 2).

The median log amplitudes were greater in the sitting compared to lying posture in all 9 eyes when the estimated CSF pressure difference was greater than 3.9 mmHg (Fig. 1). This reached statistical significance in 7 eyes (max *p* = 0.0015, Fig. 1). Multivariate linear modelling demonstrated a significant posture induced difference between log amplitudes in both eyes of subjects: B, C and F (Fig. 1), with maximum *p* value = 0.0015. The estimated CSF pressure differences for these subjects were 4.97 mmHg or greater. Similarly, when both eye data was grouped together, the same subjects with an estimated CSF pressure difference of 4.97 mmHg or greater had significant posture induced increase in log amplitudes (all *p* < 0.0001, Fig. 2) going from lying to sitting.

## Discussion

### Postural Changes

The venous pulse amplitude in this study increased in subjects moving from a lying to sitting posture proportionally with the physiologically estimated CSF pressure difference. The venous pulse amplitude is known to be influenced by CSF pressure changes and therefore it is possible that these changes directly reflect changes in CSF pressure. An increase in pulse amplitude was detected in all eyes with an estimated CSF pressure change of 3.9 mmHg or more with statistically significant change detected in all eyes with pressure changes of 5 mmHg or more. This non-invasive technique may detect a change of 5 mmHg or more based upon the differences in amplitudes observed with postural change (Figs 1 and 2). This is similar to the 95% Bland Altman confidence intervals of ±6 mmHg observed using retinal vein PPG to estimate CSF pressure^14^. The technique described here uses all the amplitude data from the hemiretinal veins, whereas the technique described to estimate CSF pressure uses only the maximum venous pulse amplitude values.

### Possible interactive factors do exist

An alternative interpretation is that the changes in venous pulse amplitude reflect changes in venous pressure around the head caused by the postural shifts. CSF pressure and cranial venous pressure are so closely linked^10,15^. that separating the two would be challenging. However, intracranial venous pressure just upstream of the large venous sinuses is usually assumed to be equivalent to CSF pressure^16^. Therefore, this technical distinction may not affect the utility of this technique for assessing CSF pressure change and so interpretation of likely CSF pressure changes will likely not be affected.

Another possibly confounding factor is that increased pulse blood pressure can increase venous pulsation^17^. In this study we found a slight trend towards a reduction in pulse pressure when sitting which was not statistically significant. It is worth noting that a reduction in pulse pressure would tend to cause a reduction in venous pulsation, the opposite of what was predicted and observed.

### Possible application

Our zero retinal vein pulsation amplitude extrapolated model has a measured accuracy of ±6.0 mmHg and is applicable at higher CSF pressure ranges, from 7 to 33 mmHg^14^. This is partly dependent upon the baseline IOP, with a lower IOP allowing the detection of lower CSF pressure with more accuracy. It is likely that CSF pressure estimation accuracy improves as CSF pressure rises, which improves the utility of this device for detecting CSF pressure elevation.

A small system producing these types of photoplethysmographic measurements could be used in outer-space to monitor CSF pressure fluctuations. Elevations in CSF pressure may be detected prior to the development of clinical signs of raised CSF pressure. This could be useful especially when papilloedema, the commonest sign, may result in impairment of optic nerve function when present over long periods. Additionally, interventions which may mitigate CSF pressure elevation or its effects on the optic nerve could possibly be assessed in the restricted environment of a space station or vehicle.

This experiment has several limitations including the small sample size of 7 subjects. The modified photo slit lamp is large and can’t easily accommodate different postures, which is a significant restriction of this approach. This limited us to a face forward position for recording all measurements. However, despite this limitation we were able to detect pulse amplitude variation in subjects undergoing the limited postural change that the system allowed. We are working to develop a hand-held system to allow true tilt table experiments to be performed. We did not perform invasive measurements of CSF pressure so we do not know the actual CSF pressure for comparison with our amplitude measures, which is a significant limitation. We used Qvarlander’s model 1 technique for estimating CSF pressure from the hydrostatic indifferent point, which is known to have an error standard deviation of 3 mmHg and so is likely to add to the error in calculating difference of estimated CSF pressure^10^. Another possible limitation of the technique is that most astronauts will exhibit papilloedema in microgravity, which may affect these measurements. In previous work^14^ we described the linearity (*r* = 0.91 with slope 1.0) and accuracy of using retinal venous pulse amplitude measures for non-invasively estimating CSF pressure in a range of subjects having invasively measured CSF pressure from 2 to 31 mmHg, and with a mix of normal optic disk morphology at lower CSF pressure to papilloedema at higher CSF pressure. In that study the slope of the relationship between estimated CSF pressure and invasively measured CSF pressure remained at 1.0 throughout the CSF pressure range, suggesting that the presence of papilloedema did not contribute to additional changes in pulse amplitudes.

## Methods

### Subjects

**Fig. 3:**
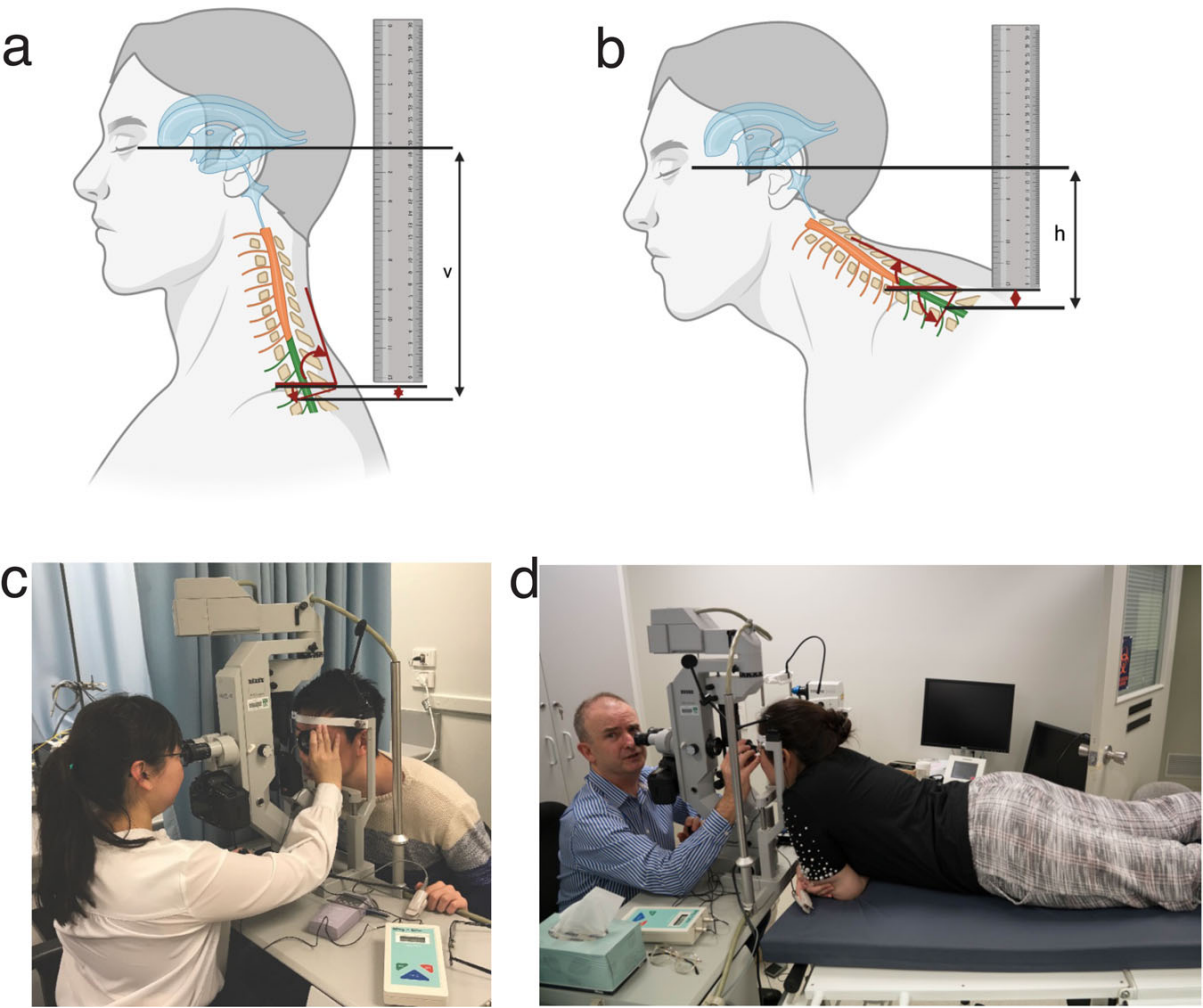
Estimating CSF pressure difference from the hydrostatic indifferent point in the two postures. Schematic diagram illustrating how the vertical height between the eye and the hydrostatic indifferent point at T2 was calculated in the 2 postures: vertical (v, **a**) and horizontal (h, **b**) and how trigonometric calculation of the depth difference to the spinal cord induced by vertebral body angulation was derived (red arrows), to add to the raw height measured. The actual experimental set-up and posture for the modified photoplethysmography using ophthalmodynamometry while sitting (**c**) and lying (**d**) is also illustrated. Consent was obtained from the three participants featured in Fig. 3.

The use of human subjects for the photoplethysmography (PPG) measurements was approved by Belberry Human Research Ethics Committee under permit number 2015-11-756-A-2, in accordance with the declaration of Helsinki and in compliance with National Health and Medical Research Council guidelines for clinical trials. Data collection was performed at the Lions Eye Institute (Nedlands, Western Australia) in accordance with relevant guidelines and regulations. Informed written consent was obtained from all participants. Subjects in Fig. 3 are all authors of this paper and gave consent for their images to be published. All measurements were obtained using standard clinical protocols for equipment cleaning and best practice following Lions Eye Institute (WA, Australia) COVID guidelines.

### Measurements

The PPG technique undertaken in this paper is described in more detail^12,18^. In brief, it allows the pressure gradient across the optic nerve to be altered while recording vessel pulsation, which is amplified when the IOP starts to exceed CSF pressure because of the non-linear venous wall response to transmural pressure. All patients underwent a full ophthalmic examination including IOP measurement and dilation with retinal examination to exclude signs of raised CSF pressure (papilloedema) and other pulsation altering pathologies such as central retinal vein occlusion and glaucoma. Intraocular pressure measurement was performed using Goldmann applanation tonometry in each posture. Additionally, blood pressure was measured using automated sphygmomanometry in each posture with the cuff at heart level. Ophthalmodynamometry was performed using a Meditron ophthalmodynamometer (Meditron, Volklingen, Germany, Fig. 3), which in principle consists of a three-mirror Goldmann lens attached to a ring force transducer allowing continuous force output. The calibration to calculate induced IOP was baseline IOP + 0.89 x force output^19^.

All subjects were examined in 2 postures: sitting on a chair and lying on a hospital bed. While on the bed, subjects were required to lie horizontal but with their head in the vertical position for PPG, supporting their chest on their forearms. This was a significant limitation where the supine position was not truly horizontal. The reason for this is that the PPG recording system comprises a 100 kg modified photo-slit lamp system which is vertical and cannot be inclined. For video-photography, all subjects had a pulse oximeter (Nellcor N65, Covidien, Mansfield, MA) placed on their right index finger with the audible oximeter ‘beep’ recorded during photography. They all positioned at the slit lamp camera (Carl Zeiss, Germany, Fig. 3) while video recordings were taken at 25 fps (Canon 5D mark III, Canon Corp, Japan). At least 3 consecutive cardiac cycle length recordings were taken at each IOP setting with pressure being applied via the Goldmann lens in approximately 5 mmHg steps from baseline to 45 mmHg.

### Pulsation amplitude calculations

For each IOP setting a 3 cardiac cycle length of video was cropped out and all frames extracted. The green colour channel video frames were spatially aligned, and a harmonic regression model fitted to the (mean) intensity time series in 5×5 pixel neighbourhoods. This in turn was used to determine the pulse amplitude for all points across the optic disc. IOP and distance from the disc centre in millimetres were recorded for each point in the vessel that generated a valid pulse amplitude. The periodic component of the harmonic regression model includes the first two terms (harmonics) of the Fourier series sine-cosine representation. The most significant component is the cosine component of the primary series and so the degree of fit for this component was used to judge quality of the harmonic fit. Only points with a primary harmonic cosine fit probability value less than 0.0001 were included in the analysis. Consistent with our previous work we used data from the inferior and superior hemiretinal veins^14,20^. The amplitude values were measured in arbitrary units using a derivation from the Beer-Lambert law relating amplitude to the negative log of intensity^21^. The retinal veins were identified manually on a corresponding image allowing retinal vein amplitude values to be extracted. The vein analysis extent was limited to data from within the central 0.4 mm of disc centre to use amplitude measurements with higher signal-to-noise ratio.

### Presumed CSF pressure difference calculation

We attempted to identify the upper section of the external jugular venous collapse, however this was not possible in the supine posture and difficult in the sitting posture. So, we adopted the CSF hydrostatic indifferent point technique (Qvarlander method 1)^10^. From work by Magnaes^9^ it is known that the average position of the CSF hydrostatic indifferent point is at T2. This is the single point along the CSF system which maintains the same pressure in the face of postural changes. One can locate T2 knowing that it is the spinous process directly below T1 a commonly used anatomical landmark. As shown in Fig. 3, while each subject was in the sitting (A) and horizontal (B) postures we used a protractor to measure the vertebral angulation from horizontal at the level of T2 (upper red curved arrow). The tilt of the vertebra was noted (Fig. 3, lower red curved arrow). Knowing that the mean T2 spinous process length was 3.0 cm^22^, the vertical drop from the T2 spinous process to the vertebral canal was calculated (Fig. 3, small red arrowheads). This was added to the vertical height from T2 to the lateral canthus measured using a ruler and spirit level to calculate the hydrostatic pressure drop from the eye to T2. By calculating the difference in vertical T2 to eye height between the vertical (v) and horizontal (h) postures one could roughly assume that this approximated the CSF pressure change in centimetres of water which was then converted to millimetres of Mercury. This was termed the estimated CSF pressure difference.

### Statistics

All results are presented with the mean and standard deviation (sd) unless otherwise specified. Pulsation amplitude distributions were non-normal. We have shown that a logarithmic transform (log_10_) normalises their distribution and so the logarithm of the amplitude was used in all statistical comparisons and in the graphical presentation^13^.

The major outcome sought was whether a relationship existed between posture and pulse amplitude. Additionally, we were interested in knowing whether a larger estimated CSF pressure difference was associated with greater increase in pulse amplitude. The major outcome/response variable was log Fourier amplitude (referred to as amplitude). The explanatory variables were posture, distance from the optic disc centre and the induced IOP. Pulse blood pressure was also tested as a potential explanatory variable. Many measurements were taken from each vessel at each IOP pressure setting from each eye. To account for inter-correlation between these multiple measurements a linear mixed model was used with a nested random effects structure where superior or inferior vein position was nested in right or left eye and patient’s identity. To test our second question we used a similar linear mixed model but introduced an interaction term between posture and estimated CSF pressure difference to test whether a greater intracranial pressure difference in sitting posture resulted in a greater pulse amplitude.

Pearson’s correlation coefficient was used to compare subject mean amplitudes with estimated CSF pressure difference. We were interested to see if the distribution of amplitudes were significantly different between the two postures within individuals. When comparing amplitudes from an individual we used a linear mixed model with amplitude as the response variable, posture, distance along vessel and IOP as explanatory variables, with right or left eye as the random factor. When comparing amplitudes from an eye we used a linear model with amplitude as the response variable, posture, distance along vessel and IOP as explanatory variables.

## Data Availability

The dataset to interpret, replicate and build upon the methods or findings reported in this article are available from the corresponding author upon reasonable request.

## Code availability

The code to interpret, replicate and build upon the methods or findings reported in this article are available from the corresponding author upon reasonable request.
